# Electrophysiological and histological comparisons of left bundle branch pacing vs. left ventricular septal pacing in canine self-controlled models

**DOI:** 10.1093/europace/euag267

**Published:** 2026-09-21

**Authors:** Jie Zhang, Fan Zhang, Shaobo Fan, Le He, Dongyan Wu, Fengmin Lu, Jing Xu, Jun Ding, Yingchuan Yan, Fengqi Xuan, Wenjuan Zhang, Wei Ma

**Affiliations:** Department of Cardiology, Clinical School of Thoracic, Tianjin Medical University, No. 70 Xi'an Road, Heping District, Tianjin 300051, China; Department of Cardiology, Chest Hospital, Tianjin University, No. 70 Xi'an Road, Heping District, Tianjin 300051, China; Department of Cardiology, Clinical School of Thoracic, Tianjin Medical University, No. 70 Xi'an Road, Heping District, Tianjin 300051, China; Department of Cardiology, Chest Hospital, Tianjin University, No. 70 Xi'an Road, Heping District, Tianjin 300051, China; Department of Cardiology, Clinical School of Thoracic, Tianjin Medical University, No. 70 Xi'an Road, Heping District, Tianjin 300051, China; Department of Cardiology, Chest Hospital, Tianjin University, No. 70 Xi'an Road, Heping District, Tianjin 300051, China; Department of Cardiology, Clinical School of Thoracic, Tianjin Medical University, No. 70 Xi'an Road, Heping District, Tianjin 300051, China; Department of Cardiology, Chest Hospital, Tianjin University, No. 70 Xi'an Road, Heping District, Tianjin 300051, China; Department of Cardiology, Clinical School of Thoracic, Tianjin Medical University, No. 70 Xi'an Road, Heping District, Tianjin 300051, China; Department of Cardiology, Chest Hospital, Tianjin University, No. 70 Xi'an Road, Heping District, Tianjin 300051, China; Department of Cardiology, Clinical School of Thoracic, Tianjin Medical University, No. 70 Xi'an Road, Heping District, Tianjin 300051, China; Department of Cardiology, Chest Hospital, Tianjin University, No. 70 Xi'an Road, Heping District, Tianjin 300051, China; Department of Cardiology, Clinical School of Thoracic, Tianjin Medical University, No. 70 Xi'an Road, Heping District, Tianjin 300051, China; Department of Cardiology, Chest Hospital, Tianjin University, No. 70 Xi'an Road, Heping District, Tianjin 300051, China; Department of Cardiology, Clinical School of Thoracic, Tianjin Medical University, No. 70 Xi'an Road, Heping District, Tianjin 300051, China; Department of Cardiology, Chest Hospital, Tianjin University, No. 70 Xi'an Road, Heping District, Tianjin 300051, China; Department of Cardiology, Clinical School of Thoracic, Tianjin Medical University, No. 70 Xi'an Road, Heping District, Tianjin 300051, China; Department of Cardiology, Chest Hospital, Tianjin University, No. 70 Xi'an Road, Heping District, Tianjin 300051, China; Department of Cardiology, Clinical School of Thoracic, Tianjin Medical University, No. 70 Xi'an Road, Heping District, Tianjin 300051, China; Department of Cardiology, Chest Hospital, Tianjin University, No. 70 Xi'an Road, Heping District, Tianjin 300051, China; Department of Cardiology, Tianjin Medical University General Hospital, No. 154 Anshan Road, Heping District, Tianjin 300052, China; Department of Cardiology, Clinical School of Thoracic, Tianjin Medical University, No. 70 Xi'an Road, Heping District, Tianjin 300051, China; Department of Cardiology, Chest Hospital, Tianjin University, No. 70 Xi'an Road, Heping District, Tianjin 300051, China

**Keywords:** Left bundle branch area pacing, Left bundle branch pacing, Left ventricular septal pacing, Canine model, Left bundle branch capture, Self-controlled study

## Introduction

Growing clinical and mechanistic data demonstrate that left bundle branch area pacing (LBBAP) yields superior cardiac functional improvement in heart failure patients.^1–3^ Confirmation of left bundle branch (LBB) capture is essential for LBBAP to exert physiological therapeutic effects.^4,5^ Although diagnostic criteria for LBB capture have evolved from simple electrocardiogram(ECG) markers to multi-dimensional assessment systems, interindividual differences in cardiac anatomy, pathology, myocardial function, and electrophysiology limit their general applicability.^6,7^ Systematic animal studies investigating electrophysiological differences between LBBP and left ventricular septal pacing (LVSP) remain scarce. Rigorous self-controlled comparisons with histological lead-position validation remain largely lacking. We therefore established a within-subject canine LBBAP model capturing vs. non-capturing the conduction system, aiming to minimize individual confounders and identify reliable markers of LBB capture.

## Materials and methods

Eight adult canines (25–35 kg) were included. All animal protocols were ethically approved and performed per standard laboratory animal guidelines. General anaesthesia and mechanical ventilation were maintained to ensure high-quality three-dimensional electroanatomic mapping (3-D EAM) and intracardiac echocardiography (ICE).

LBBP and LVSP were delivered sequentially in each animal. Pacing leads were implanted into the subendocardium of the high left-ventricular septum under fluoroscopic, 3-D EAM, and ICE guidance. After successful LBBP lead implantation, the LVSP lead was positioned as close as possible to the LBBP site following the identical implantation technique. The lead helix was advanced incrementally under constant low pacing outputs (2 V/0.5 ms).

Successful LBBP met all three criteria: (i) paced QRS complex(QRS) with right bundle branch block morphology in lead V1; (ii) short, stable stimulus-to-left ventricular activation time (Stim-LVAT) at low and high pacing outputs (V6 recording); (iii) distinct local LBB potential at the pacing site, with consistent intervals between stimulus-to-retrograde His potential (Stim-Retro-His) and intrinsic His-LBB potential. LVSP was defined as exclusive septal myocardial capture without LBBP features, presenting an atypical terminal R wave in V1.

Unipolar pacing was delivered for both modalities, with continuous recording of 12-lead ECG and intracardiac electrograms. Stepwise decremental S1-S1 burst pacing measured LBB and septal-myocardial effective refractory periods (ERPs). Gross anatomical observation and histological staining were conducted to confirm the lead position relative to the left-ventricular conduction system.

SPSS 27 was used for statistical analyses. Paired continuous variables were assessed via paired *t*-test or Wilcoxon signed-rank test; paired categorical variables via McNemar’s test. The significance threshold was *P* < 0.05.

## Results

Eight dogs were enrolled in this study. Two were excluded for failure to obtain stable LBB capture after repeated lead repositioning, leaving six animals for sequential LBBP and LVSP evaluations. Representative electrode positions and ECG findings for both pacing modalities under multimodal imaging guidance are shown in *Figure 1A*.

**Figure 1:**
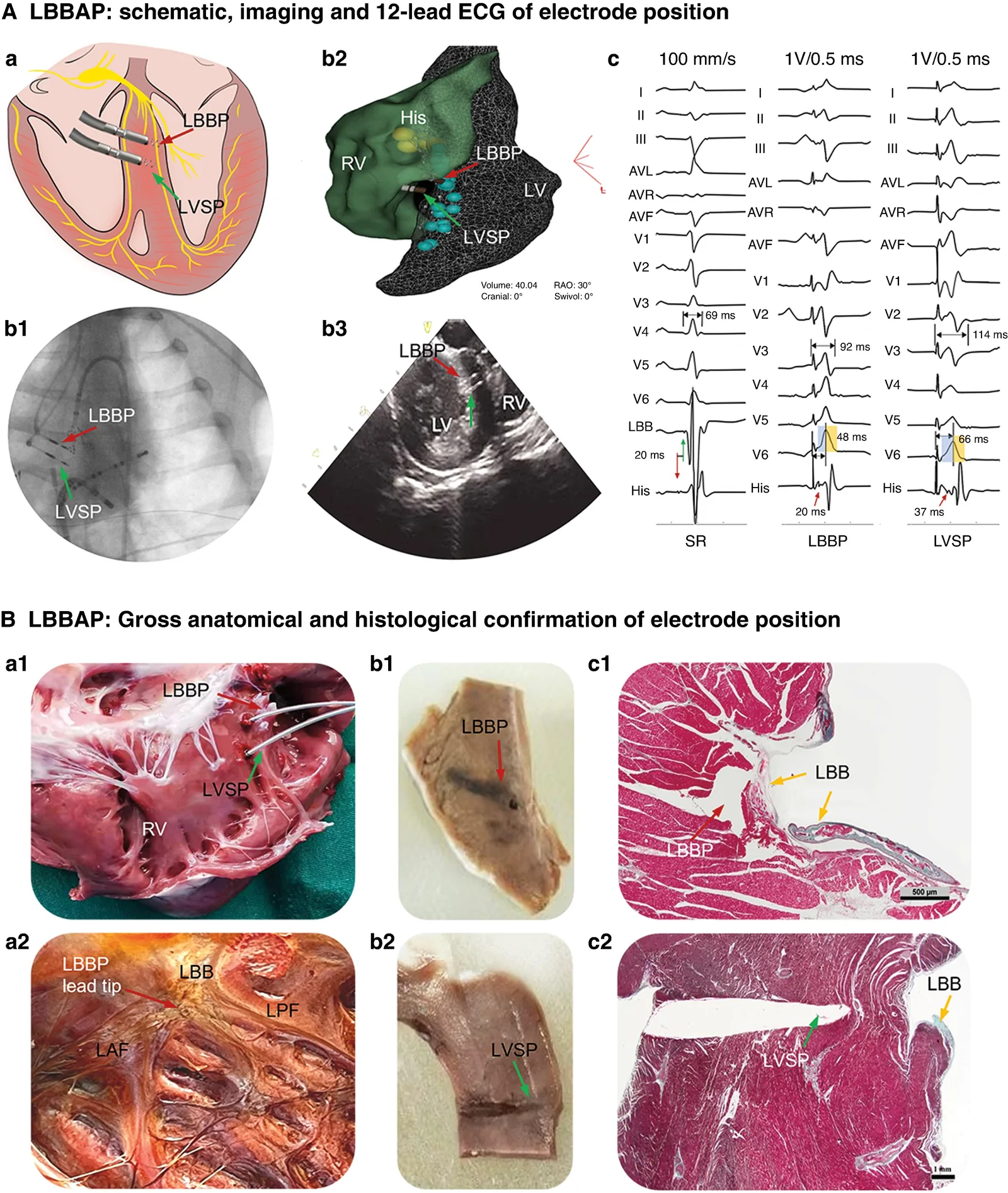
LBBP vs. LVSP: multiplanar visualization of electrode tip locations and paced ECG characteristics. Panel *Aa* schematically shows the theoretical lead tip positions of LBBAP. Panels *Ab1–Ab3* display the post-implant LBBAP lead tip positions visualized via fluoroscopy, 3-D mapping system, and ICE. Panel *Ac* presents paced ECG features for LBBP and LVSP. A distinct LBB potential is observed during LBBP, and retrograde His potentials are recorded under both pacing modalities. Panels *Ba1* and *Ba2* illustrate the initial and final screw-in positions of the LBBAP leads across the right-to-left ventricular septum. Panels *Bb1* and *Bb2* delineate the electrode penetration tract and distal tip position within the left ventricular septum. Panels *Bc1* and *c2* show histological images illustrating the distance from electrode tips to the left-sided conduction system under both pacing modalities. Notably, the LBBP electrode tip makes direct contact with the left conduction system. Abbreviations: SR, sinus rhythm; LBBAP, left bundle branch area pacing; LBBP, left bundle branch pacing; LVSP, left ventricular septal pacing; LAF, left anterior fascicle; LPF, left posterior fascicle; ICE, intracardiac echocardiography.

LBBP and LVSP exhibited distinct electrophysiological features. Retrograde His potentials were recordable on all 12 paced leads. Compared with LVSP, LBBP yielded shorter stimulus-to-QRS end intervals (91.1 ± 4.7 ms vs. 111.5 ± 4.6 ms), Stim-LVAT (47.5 ± 2.4 ms vs. 64.5 ± 3.3 ms) and Stim-Retro-His intervals (18.9 ± 2.8 ms vs. 36.1 ± 1.7 ms), alongside longer V6−V1 interpeak intervals (33.8 ± 1.5 ms vs. 25.9 ± 3.2 ms, all *P* < 0.001). During incremental lead screwing into the septum, Stim-LVAT dropped abruptly by 13.5 ± 1.9 ms in LBBP, whereas no shift was observed with LVSP. The V6 upstroke/downstroke ratio showed complete separation between the two pacing modalities: values were persistently <1 for LBBP and >1 for LVSP. Furthermore, septal myocardium exhibited a markedly shorter ERP than LBB tissue (258.4 ± 11.7 ms vs. 310.5 ± 15.5 ms, *P* < 0.001).

Hearts were explanted to confirm lead positioning. Panel B a1 illustrates screw-in sites of LBBAP leads on the right ventricular septum. Gross inspection demonstrated intense dark brown staining of the LBB and Purkinje network across the left-ventricular endocardium. The LBBP lead was embedded in the subendocardium overlying proximal LBB fascicles, with its helix faintly visible (Panel B a2). Histological sections of the interventricular septum clearly delineate the lead tract, penetration depth, and distal tip location of LBBAP leads. In all six canines, LBBP lead tips formed a 60°–90° angle with the left-ventricular endocardium, closely apposing or attached to the LBB trunk or proximal branches (Panels B b1, c1). LVSP lead tips rested adjacent to the left-ventricular endocardium and remained distant from the conduction system (Panels B b2, c2). Mean implantation depth was significantly greater for LBBP (12.0 ± 1.4 mm vs. 9.8 ± 1.2 mm; *P* = 0.007).

## Discussion

Clinically, rapid, reliable discrimination of LBBP vs. LVSP remains challenging.^8^ Our self-controlled study design minimizes confounding variables, thereby bolstering data reliability. Multimodal evidence—comprising electrophysiological, ECG, and histological findings—confirmed the criteria for identifying true LBB capture.

Interspecies and body weight variations alter absolute values of core LBB capture indices compared with prior studies.^9,10^ However, an abrupt ≥10 ms reduction in Stim-LVAT during electrode screw-in and a paced V6 QRS upstroke-to-downstroke ratio <1 each outperform standalone absolute ECG metrics for discriminating LBB capture, offering superior diagnostic accuracy and clinical utility. This is the first histological validation of these rapid ECG criteria for LBB capture in LBBAP.

A retrograde His potential is conventionally deemed a hallmark of LBB capture and rarely recorded during LVSP.^5,9^ Nevertheless, this potential was recorded in all animals under both pacing modalities, demonstrating that this marker cannot serve as a standalone criterion. Instead, a shorter Stim-retro-His interval (cut-off of 25 ms, 100% specificity in this study) yields superior clinical discriminative value.

Limitations include the relatively small sample size and interspecies differences in LBBP-related anatomy and conduction velocity. In addition, LVSP leads were positioned more distally than LBBP leads, potentially confounding the electrophysiological findings. Even though the pronounced difference in conduction velocity between the LBB and adjacent myocardium outweighs the influence of this small spatial offset within the high septal region, these findings should be interpreted with caution.

In conclusion, this self-controlled LBBP/LVSP protocol minimized interindividual variability. An abrupt Stim-LVAT reduction ≥10 ms during lead screwing, a paced V6 QRS upstroke-to-downstroke ratio <1, and a Stim-retro-His interval ≤25 ms enable rapid, reliable discrimination of true LBB capture.

## Data Availability

Data are available on request to the corresponding author.
